# Galectins-1 and-3 Increase in Equine Post-traumatic Osteoarthritis

**DOI:** 10.3389/fvets.2018.00288

**Published:** 2018-11-20

**Authors:** Heidi L. Reesink, Alan J. Nixon, Jin Su, Sherry Liu, Ryan M. Sutton, Sabine Mann, Ashlee E. Watts, Ryan P. Peterson

**Affiliations:** ^1^Department of Clinical Sciences, College of Veterinary Medicine, Cornell University, Ithaca, NY, United States; ^2^Department of Population Medicine and Diagnostic Sciences, College of Veterinary Medicine, Cornell University, Ithaca, NY, United States

**Keywords:** inflammatory arthritis, cartilage, synovium, chondrocyte, synoviocyte, horse, rheumatoid arthritis

## Abstract

Galectins are potent regulators of cell adhesion, growth and apoptosis in diverse cell types, including chondrocytes and synovial fibroblasts. Elevations in synovial fluid galectin-3 have been observed in rheumatoid arthritis, juvenile idiopathic arthritis and experimental inflammatory arthritis in animal models, whereas galectin-1 is thought to be protective. Less is known about galectins-1 and-3 in osteoarthritis (OA). Therefore, the purpose of this study was: (1) to determine whether galectin-1 and-3 synovial fluid concentrations and synovial membrane and cartilage histochemical staining were altered following osteochondral injury in an experimental equine osteoarthritis (OA) model and (2) to measure galectin-1 and-3 mRNA expression and synovial fluid concentrations in naturally occurring equine carpal OA. Synovial fluid galectin-1 and-3 concentrations were quantified using custom ELISAs in two research horse cohorts undergoing experimental OA induction (*n* = 5 and 4) and in a cohort of horses with naturally occurring carpal OA (*n* = 57). Galectin mRNA expression in synovial membrane and cartilage tissue obtained from carpal joints of horses with naturally occurring OA was measured using RT-qPCR, and galectin immunostaining was assessed in synovial membrane and osteochondral tissues in the experimental model (*n* = 5). Synovial fluid galectin-1 and-3 concentrations increased following experimental carpal osteochondral fragmentation. Cartilage galectin-1 mRNA expression increased with OA severity in naturally occurring disease. The superficial zone of healthy articular cartilage stained intensely for galectin-3 in sham-operated joints, whereas galectin-1 staining was nearly absent. Chondrocyte galectin-1 and-3 immunoreactivity increased following cartilage injury, particularly in galectin-1 positive chondrones. Galectins-1 and-3 are present in healthy equine synovial fluid and increase following post-traumatic OA. Healthy superficial zone chondrocytes express galectin-3, whereas galectin-1 chondrocyte staining is limited predominantly to chondrones and injured cartilage. Further work is needed to clarify the functions of galectins-1 and-3 in healthy and OA joints.

## Introduction

Galectins are potent regulators of cell adhesion, growth, and apoptosis in diverse tissues and organs, including synovial joints. Galectin-1 and galectin-3 are expressed in synovial fibroblasts, articular chondrocytes, and hypertrophic growth plate chondrocytes (1–3). Synovial fibroblasts express higher levels of galectin-1 and-3 as compared to articular chondrocytes (4), and intracellular galectin-3 promotes chondrocyte survival in both articular and hypertrophic chondrocytes (5, 6).

Associations between increased galectin-3 in synovial fluid, synovial tissues, and sera of human patients with inflammatory arthritis have been observed in several studies (7–9), and strong expression of galectin-3 at sites of joint destruction has led authors to suggest that galectin-3 plays a role in rheumatoid arthritis (RA) pathogenesis (10). Some authors have even proposed that galectin-3 may be a potential therapeutic target for RA (11, 12). Conversely, decreased galectin-1 concentrations and increased anti-Gal-1 antibodies have been detected in RA patients (13). Galectin-1 expression is downregulated and galectin-3 expression is upregulated in synovial tissue from patients with juvenile idiopathic arthritis (8). Most rodent experimental models of inflammatory arthritis suggest that galectin-1 is protective (14–17), whereas galectin-3 promotes joint inflammation (16, 18). For example, galectin-1 knockout (KO) mice develop earlier onset and more severe collagen-induced arthritis (14), and galectin-3 KO mice have reduced inflammation and bone erosion in response to antigen-induced arthritis as compared to wild-type mice (18). In addition, while both recombinant protein and genetic delivery of galectin-1 are protective in rodent models of collagen-induced arthritis (15–17), administration of galectin-3 shRNA protects rodents from collagen-induced arthritis (16). Classification of galectin-3 as a driver or inhibitor of inflammatory arthritis is likely affected by its intracellular or extracellular localization (6, 19), with rodent knockout models emphasizing intracellular galectin signaling.

The role of galectins in osteoarthritis (OA) and post-traumatic osteoarthritis (PTOA) is not well understood. What little is known about galectin-1 and-3 synovial fluid levels or synovial membrane localization in human OA is derived from studies where OA patients were used as a comparison group to RA patients (7). Galectin-1 and-3 levels have not been evaluated in synovial fluid from healthy human patients, with the exception of a “healthy control” group in two studies in which synovial fluid was obtained pre-operatively from patients with knee trauma or meniscal tears (7, 20). Lectin/galectin staining in human OA cartilage has revealed increased galectin-1 and galectin-3 chondrocyte immunostaining at sites of cartilage damage (21–23), with increasing galectin-1 positivity correlated with cartilage Mankin scores (21, 23). Galectin-1 and-3 mRNA was expressed in human OA chondrocytes at higher levels than galectins-2, 4, 7, 8, and 9; however, levels of galectin expression in healthy chondrocytes were not studied (23). Investigation of *in vitro* signaling pathways in human OA chondrocytes revealed that both galectins-1 and-3 promote an inflammatory gene signature, at least in part through their role as upstream NF-κB signaling effectors (21, 24). On the other hand, galectin-3 KO mice demonstrate increased cartilage damage in a mono-iodoacetate injection model of OA (6), and galectin-3 KO mice also demonstrate increased bone resorption and accelerated trabecular bone loss as compared to wild-type mice (25), suggesting a protective role for galectin-3 in OA.

To the authors' knowledge, galectins have only been evaluated in animal models of inflammatory arthritis and not in PTOA models. Therefore, critical gaps in knowledge include understanding: (1) how synovial fluid galectin-1 and-3 concentrations change over time following joint injury, (2) how galectin-1 and-3 mRNA expression and synovial fluid levels differ in healthy as compared to OA joints, and (3) whether galectin-1 and-3 immunostaining differs between healthy and OA cartilage in PTOA. Horses are athletic animals that commonly develop PTOA in the course of their performance careers (26). Synovitis, cartilage impact injury, osteochondral fragmentation and subchondral bone injury are common in the high-motion carpal joints (27). Because PTOA in horses can better recapitulate certain aspects of human PTOA pathogenesis than chemically induced models in rodents (28), we chose to evaluate galectins-1 and-3 in the equine model. Synovial fluid and articular tissues were obtained from horses with naturally occurring OA, and the carpal osteochondral fragment high-speed treadmill exercise model of OA was used to evaluate serial changes in synovial fluid galectins and cartilage immunohistochemistry. Biochemical, histologic, and inflammatory changes are well-characterized in the equine carpal osteochondral fragment model of OA (27, 29, 30), and this model is commonly used to test the therapeutic effects of intra-articular or systemic OA therapies (27, 31).

Therefore, the objectives of this study were to: (1) compare galectin-1 and-3 mRNA expression and synovial fluid concentrations in healthy and OA joint tissues from horses with naturally occurring OA, and (2) to determine whether galectin-1 and-3 serial synovial fluid concentrations and galectin immunostaining were altered following osteochondral injury in an experimental equine OA model.

## Materials and methods

### Ethics statement

All experimental protocols were approved by the university Institutional Animal Care and Use Committee (protocol numbers: 2011-0027 and 2012-0097). All sample collection was performed following humane euthanasia of horses using sodium pentobarbital or obtained from discarded tissues following arthroscopic surgery of horses with informed consent from owners.

### Equine carpal osteochondral fragment model

Synovial fluid samples collected from two distinct equine experimental cohorts (*n* = 5 and *n* = 4) undergoing carpal fragmentation were used to measure serial galectin-1 and-3 concentrations. Synovial membrane biopsies and osteochondral tissues were collected from the first cohort (*n* = 5) for immunohistochemistry following euthanasia on day 70 post-fragmentation. Analysis of synovial fluid lubricin concentrations and lubricin immunostaining has previously been reported in the first cohort (32). Horses in both cohorts were subjected to carpal osteochondral fragmentation in one randomly assigned joint, while the opposite joint served as a sham-operated control. Two weeks post-operatively, horses commenced a high-speed treadmill exercise program 5 times weekly, continuing throughout the study duration of either 70 or 75 days. Five Thoroughbred horses (*n* = 3 females and 2 castrated males), aged 2–6 years old, were enrolled in the first cohort, and four Thoroughbred horses (*n* = 2 females and 2 castrated males), aged 2–6 years old, were enrolled in the second cohort. Experimental protocols were roughly similar between the two groups; however, the timing of synovial fluid collection and study duration differed slightly. All horses were housed in 3.65 × 3.65 m box stalls and engaged in similar treadmill exercise programs, consisting of walking (5 km/h) for 5 min, followed by trotting (16–18 km/h) for 2 min, galloping (28–32 km/h) for 2 min, and ending with 2 min of trotting (16–19 km/h) exercise performed in the morning. Synovial fluid aspirates were processed similarly, and synovial fluid supernatants were stored in aliquots at −80°C following centrifugation at 3,000x g for 5 min to pellet any cellular debris. Synovial fluid samples were collected and banked from the first cohort approximately 2 years prior to the second cohort, and all samples were frozen at −80°C for long-term storage. Synovial fluid samples from the first cohort were subjected to up to 3 freeze-thaw cycles prior to ELISA measurements, whereas samples from the second cohort were only subjected to 1 freeze-thaw cycle.

### Naturally occurring Equine OA

Synovial fluid and discarded tissues, including synovial membrane and osteochondral tissues, were harvested where available from the antebrachialcarpal (ACJ) and middle carpal joints (MCJ) of horses undergoing arthroscopic surgery at the Cornell University Equine Hospital, with informed owner consent. Each joint was assessed as healthy (grade 0) or assigned an osteoarthritis (OA) severity score of mild (1), moderate (2) or severe (3) on the basis of radiographic evidence of osteophytes, enthesiophytes, osteoproliferation, joint space narrowing or chronic fracture lines as previously described (33). Synovial fluid and tissues were also collected from horses donated for research purposes, and joint scores were assessed on the basis of radiographic and/or gross dissection findings. Thoroughbred, Standardbred or Quarter Horse females (*n* = 34), intact males (*n* = 6), or castrated males (*n* = 17) ranging in age from 2 to 13 years were included. A total of 54 and 52 synovial fluid samples were quantified using galectin-1 and-3 ELISAs, and 57 synovial membrane and 34 cartilage tissue samples were analyzed via RT-qPCR.

### Galectin-1 and-3 synovial fluid ELISA

Equine galectin-1 (GenBank ID: KY264050.1) and galectin-3 (GenBank ID: KY264051.1) were cloned, recombinantly expressed in *E. coli*, and purified using lactosyl sepharose chromatography and FPLC gel filtration as previously reported (34). Galectin ELISA antibody reactivity to recombinant equine galectin standards has previously been described (4). Both the goat anti-mouse Gal-1 antibody (AF1245; R&D Systems, Minneapolis, MN) and the goat anti-human Gal-3 antibody (sc-19280; Santa Cruz Biotechnology, Dallas, TX) were biotinylated using the Mix-n-Stain biotin antibody labeling kit (Biotium Inc., Fremont, CA), and biotinylated goat anti-mouse Gal-3 antibody (BAF1197, R&D Systems, Minneapolis, MN) was obtained from the manufacturer. Synovial fluid samples were resolved on 12% SDS-PAGE gels and probed with the biotinylated primary antibodies to confirm antibody reactivity to equine synovial fluid galectins. All 3 biotinylated primary antibodies were used at a concentration of 0.15 μg/mL in blocking buffer (3% BSA in 0.1% PBS-Tween for anti-mouse Gal-1 and anti-mouse Gal-3 antibodies; 1% Gelatin in 0.1% PBS-Tween for anti-human Gal-3). Streptavidin-HRP was applied at 4 ng/mL in 0.1% PBS-Tween for all blots.

Custom galectin-1 competitive inhibition ELISAs (4) were performed on banked equine synovial fluid samples from two cohorts of horses subjected to carpal osteochondral fragmentation. Briefly, 96-well high-binding plates (Corning Inc., Corning, NY) were coated with 1 μg/mL of goat anti-mouse Gal-1 capture antibody in sodium carbonate buffer, pH 9.6 at 4°C. After 3 rinses in 0.1% PBS-Tween, protein free blocking buffer (Thermo Fisher Scientific, Rockford, IL) was added for 1 h. Unlabeled recombinant equine galectin-1 standards (2 μg/mL to 15.6 ng/mL) were diluted in 200 ng/mL biotinylated recombinant equine galectin-1 in 0.1% PBS-Tween. Synovial fluid samples pre-diluted 1:50 in PBS were further diluted 1:1, for a final dilution of 1:100, in 200 ng/mL of biotinylated recombinant equine galectin-1. Blocking buffer was aspirated, and 100 μL of recombinant equine galectin-1 standards or synovial fluid samples were added to the plate in duplicate and incubated for 1 h at RT. Plates were rinsed in 0.1% PBS-Tween, incubated with 100 μL of streptavidin HRP for 30 min. TMB reagent was added for 10 min prior to halting the reaction with 1N H_2_SO_4_. Absorbance was measured at 450 nm with 540 nm background subtraction.

For the custom galectin-3 sandwich ELISA, 96-well plates were coated with 2 μg/mL of goat anti-human Gal-3 capture antibody (sc-19280) using similar methodology as for Gal-1. After rinsing in 0.1% PBS-Tween, protein-free blocking buffer was added for 1 h, followed by serial dilutions of recombinant equine galectin-3 standards (400 ng/mL to 1.6 ng/mL) in duplicate. Synovial fluid samples pre-diluted 1:50 in PBS were further diluted 1:1 in 1% BSA in PBS, for a final dilution of 1:100, and added in duplicate to the plate. Following 1 h incubation at RT, the plate was rinsed in 0.1% PBS-Tween, and biotinylated goat anti-mouse Gal-3 pAb (BAF1197) was added at 200 ng/mL for 1 h. Following rinsing, 100 μL of streptavidin HRP was added for 30 min prior to adding 1N H_2_SO_4_. Absorbance was measured at 450 nm with 540 nm background subtraction.

### Galectin-1 and-3 RT-qPCR

Synovial membrane and cartilage tissues were snap-frozen in liquid nitrogen and stored at −80°C for up to 3 years. The frozen tissues were crushed and ground into fine powder in liquid nitrogen with a mortar and pestle prior to isolation of RNA. Total RNA was extracted using the E.Z.N.A Tissue RNA Kit (Omega BioTek, Inc., Norcross, GA) for synovial membrane or the RNeasy Lipid Tissue Mini Kit (QIAGEN Sciences Inc., Germantown, MD) for cartilage. DNase I was added during RNA extraction to remove genomic DNA. RNA purity and concentration were assessed with a multimode plate reader (Tecan Spark^®;^ 10M, Tecan, Austria) with a NanoQuant Plate^TM^. Expression of galectin-1 and galectin-3 mRNA was quantified using the Taqman RNA-to-CT one-step kit (Applied Biosystems, Foster City, CA) and the ABI PRISM 7900 HT Sequence Detection System (Applied Biosystems, Foster City, CA). As previously reported, Primer Express Software Version 2.0 (Applied Biosystems, Foster City, CA) was used to design forward and reverse primers and probes for equine galectins-1 and-3 (4). Primer and probe sequences are listed in Table 1. Equal amounts (10 ng per reaction) of total RNA were added in a 20 μl reaction volume for all samples. The RT-qPCR reactions were all performed in duplicate. The levels of gene expression were calculated using the standard curves generated with serial dilutions of *E. coli*-expressed equine *Gal-1* and *Gal-3* as standards. All data were normalized with the housekeeping gene equine 18S rRNA. A qPCR checklist is provided to document the technical aspects of qPCR Protocols (Supplementary Material).

**Table 1:** Genes, primers, and probes for TaqMan RT-qPCR.

**Genes**	**Accession number**	**Primer sequences**	**Probe sequences**	**Amplicon size (bp)**
*EqGal-1*	KY264050.1	For: CAAGGCAGACCTGACCATCARev: TGACGGCCTCCAGGTTGA	6-FAM/CTGCCGGAT/ZEN/GGCTACTCGT TCAAGTTC/IABkFQ	77
*EqGal-3*	KY264051.1	For: TAAATTTCAACAGAGGGCATGATGRev: CAATGACTCTCCTGTTGTTCTCGTT	6-FAM/TGCCTTCCA/ZEN/CTTTAACCCG CGCTT/IABkFQ	75
*Eq 18S* rRNA	NR_046271.1	For: GGCGTCCCCCAACTTCTTRev: AGGGCATCACAGACCTGTTATTG	6-Fam/TCGAACGTCTGCCCTATCAACT TTCGAT/IABkFQ	77

### Galectin-1 and-3 immunohistochemistry in experimental Equine OA

Synovial membrane and osteochondral tissue sections from the radial carpal bone and opposing third carpal bone were stained for galectin-1 and galectin-3 using previously reported techniques (34). Briefly, osteochondral sections were fixed in 4% paraformaldehyde, de-calcified in 10% EDTA for 3 weeks and embedded in paraffin, while synovial membrane sections were embedded in paraffin immediately after fixation. Following deparaffinization, sections were treated with 1% hyaluronidase (Sigma-Aldrich, St. Louis, MO) in 20 mM sodium acetate for 30 min at 37°C, followed by 3% hydrogen peroxide for an additional 30 min. Blocking in normal rabbit serum was performed, followed by incubation with a goat anti-mouse galectin-1 antibody (AF1245; R&D) or goat anti-human galectin-3 antibody (sc-19280; Santa Cruz) at 1:100 dilution for 1 h at room temperature. After washing, sections were incubated with a biotinylated rabbit anti-goat IgG (Vectastain, Vector Labs) and immunodetected with the Vectastain ABC Kit and ImmPACT DAB reagent (Vector Labs). Negative controls were performed by omission of primary antibody. Sections were rinsed in PBS, counterstained with Harris hematoxylin, coverslipped and imaged with a 20x objective using a ScanScope (ScanScope CS0, Aperio). Images were saved as.tif files in Aperio's Image Scope software, cropped in Adobe Photoshop CC and formatted in Adobe Illustrator CC.

Synovial membrane tissue sections were imaged but not quantified due to the presence of strong galectin-1 and galectin-3 immunostaining in all sections. Osteochondral sections from the radial carpal bone and third carpal bone were scored independently by two observers. Although observers were blinded to individual animal identity, blinding to treatment group (sham-operated or OA) was not possible due to the presence of obviously injured articular cartilage within some osteochondral sections. Where possible, separate scores were assessed for injured cartilage regions vs. healthy cartilage regions within the same section. Chondrocytes within each cartilage zone (superficial, middle and deep) were assigned an immunostaining intensity score for both galectin-1 and galectin-3 where: 0–none, 1–weak, 2–moderate, and 3–strong. In addition, the percentage of galectin-1-positive chondrocytes was calculated for the entire articular cartilage section, including superficial, middle, and deep zones combined.

### Statistical analysis

To assess the effect of treatment (sham vs. OA) and time (day) on galectin concentrations in synovial fluid, galectin ELISA data were first tested for normality using a Shapiro-Wilk W test and were found to be right skewed. Log transformation was performed to achieve normality. In order to account for the hierarchical nature of the data in the experimental models, a mixed linear model was employed because each horse was repeatedly measured on each limb and each limb repeatedly over days. The fixed effects in the model included treatment (sham vs. OA), day and a treatment^*^day interaction term, and random effects included horse and individual limb nested within horse to account for the non-independence of the observations. Predefined *post hoc* comparisons of specific contrasts for each time point were performed to assess differences between sham and OA joints, with a Bonferroni correction applied based on the number of multiple comparisons to correct for the false discovery rate. Model diagnostics were performed and showed normality and homoscedasticity of residuals. Spearman correlation analysis was performed to determine associations between synovial fluid galectin-1 and galectin-3 levels and previously published lubricin ELISA data for the first experimental cohort and for the naturally occurring OA cases (32). Raw RT-qPCR and ELISA data from naturally occurring samples were log-transformed to achieve normality of the data, and data were analyzed using a one-way ANOVA with Dunnett's *post hoc* tests for multiple comparison correction, designating healthy carpal joints (OA severity = 0) as the control group. Significance was set at α = 0.05.

Immunohistochemistry scores (0 to 3 scale) were treated as ordinal categorical outcomes, and weighted kappa statistics were calculated for inter-observer agreement. Immunostaining results were assessed using Wilcoxon matched-pairs signed rank tests due to the small sample size and non-normal distribution of data. For the percentage of galectin-1 positive chondrocytes throughout the entire cartilage section, scores were treated as continuous outcomes and analyzed using Wilcoxon matched-pairs signed rank tests. All modeling and parametric analyses were performed using JMP Pro 13 software (SAS; Cary, NC), and non-parametric test statistics, kappa statistics and graphs were generated using Prism 7 (GraphPad; La Jolla, CA).

## Results

### Synovial fluid galectin protein concentrations

#### Experimental OA

**Figure 1:**
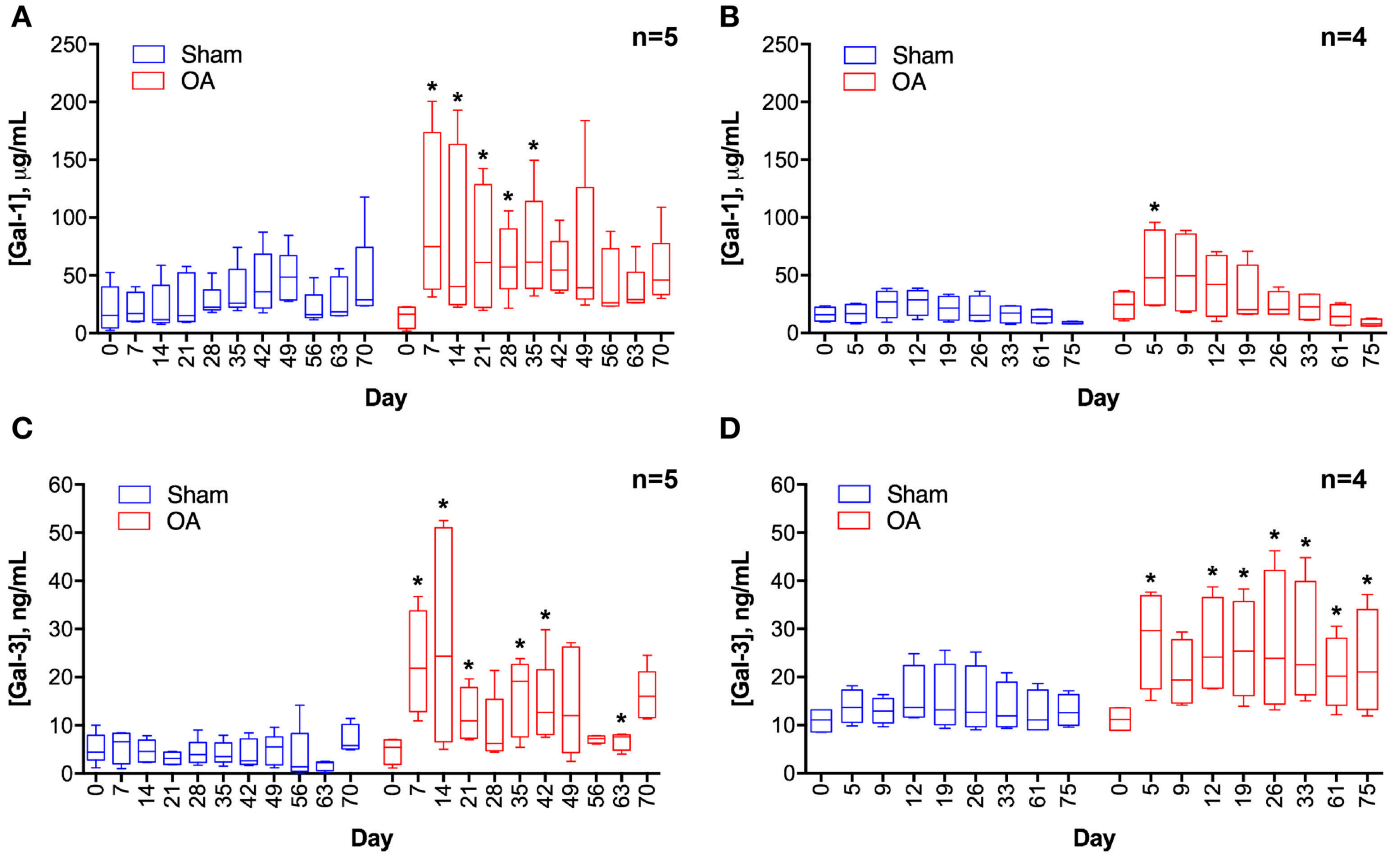
Galectin-1 **(A,B)** and galectin-3 **(C,D)** concentrations in equine synovial fluid prior to (day 0) and after arthroscopically-induced osteochondral fragmentation (OA) or sham operation (Sham). Data are displayed as box-and-whisker plots representing the first and third quartiles, median, and spread of concentrations for each serial sampling time point. ELISA data in **(A,C)** were obtained from the same cohort of horses (*n* = 5), and ELISA data in **(B,D)** were obtained from a second cohort of horses (*n* = 4). Mixed linear model derived *P*-values for the fixed effect of treatment and day were < 0.001 for models A-D, except for D where day *P* = 0.32. An interaction between treatment and day was identified for model A (treatment ^*^ day *P* = 0.0007) and C (treatment ^*^ day *P* = 0.03). Note that there is variation in the date of synovial fluid sampling between the two cohorts. Asterisks denote days where OA galectin concentrations were significantly increased as compared to Sham, ^*^ = *P* < 0.05 after Bonferroni correction.

Synovial fluid galectin-1 and galectin-3 concentrations increased following carpal osteochondral fragmentation in both experimental cohorts (Figure 1). In the carpal osteochondral fragment model, galectin-1 synovial fluid concentrations increased by up to 4-fold (median: 74.8 μg/mL vs. 16.9 μg/mL on day 7), and galectin-3 synovial fluid concentrations increased by as much as 5-fold in the OA joint as compared to the sham-operated joint (median: 24.3 ng/mL vs. 4.6 ng/mL on day 14). Galectin-1 synovial fluid concentrations were most elevated acutely after injury, whereas elevations in galectin-3 were sustained up to the end of the study duration (day 75) in one cohort of horses (Figure 1D). Galectin-3 was moderately correlated with synovial fluid lubricin (ρ = 0.47, *P* < 0.0001), whereas galectin-1 was weakly correlated with lubricin (ρ = 0.27, *P* = 0.007) (32). Galectin-1 and-3 synovial fluid levels were weakly correlated (ρ = 0.31, *P* = 0.002).

#### Naturally occurring OA

**Figure 2:**
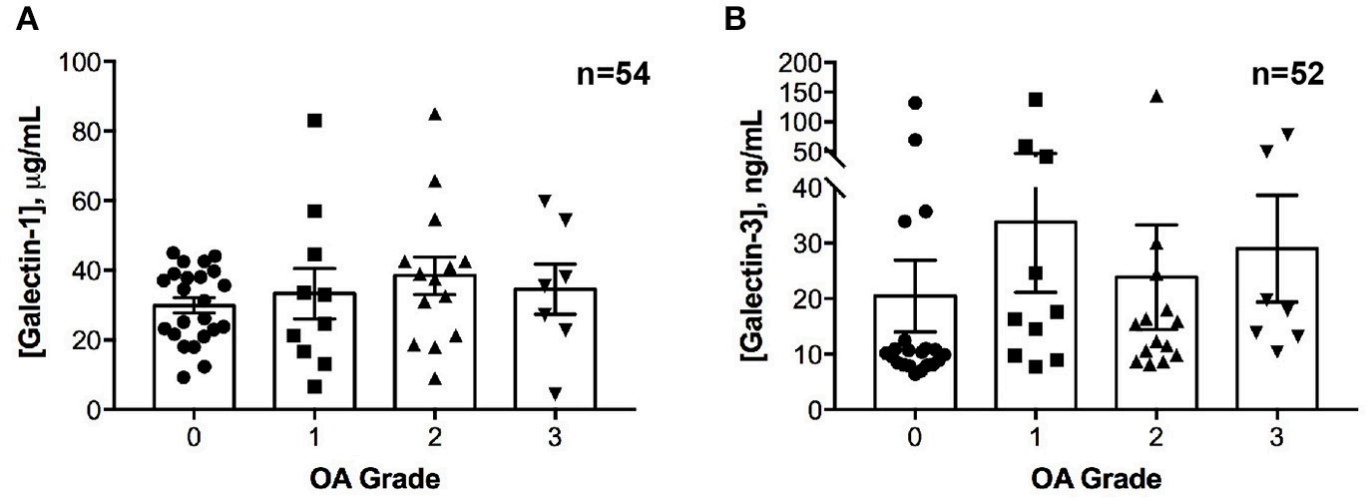
Galectin-1 and-3 concentrations in equine synovial fluid obtained from carpal joints of horses with naturally occurring osteoarthritis (OA), classified by severity as mild (1), moderate (2) or severe (3) and from healthy carpal joints (0). Individual data are shown as scatterplots, in addition to means ± S.E.M. There were no significant differences in synovial fluid galectin-1 **(A)** or galectin-3 **(B)** concentrations between healthy and OA joints.

No differences in galectin-1 or galectin-3 concentrations were detected in healthy joints as compared to joints with naturally occurring OA (Figure 2). Galectin-1 and galectin-3 synovial fluid concentrations were weakly correlated (ρ = 0.28, *P* = 0.04).

### mRNA expression in naturally occurring OA

**Figure 3:**
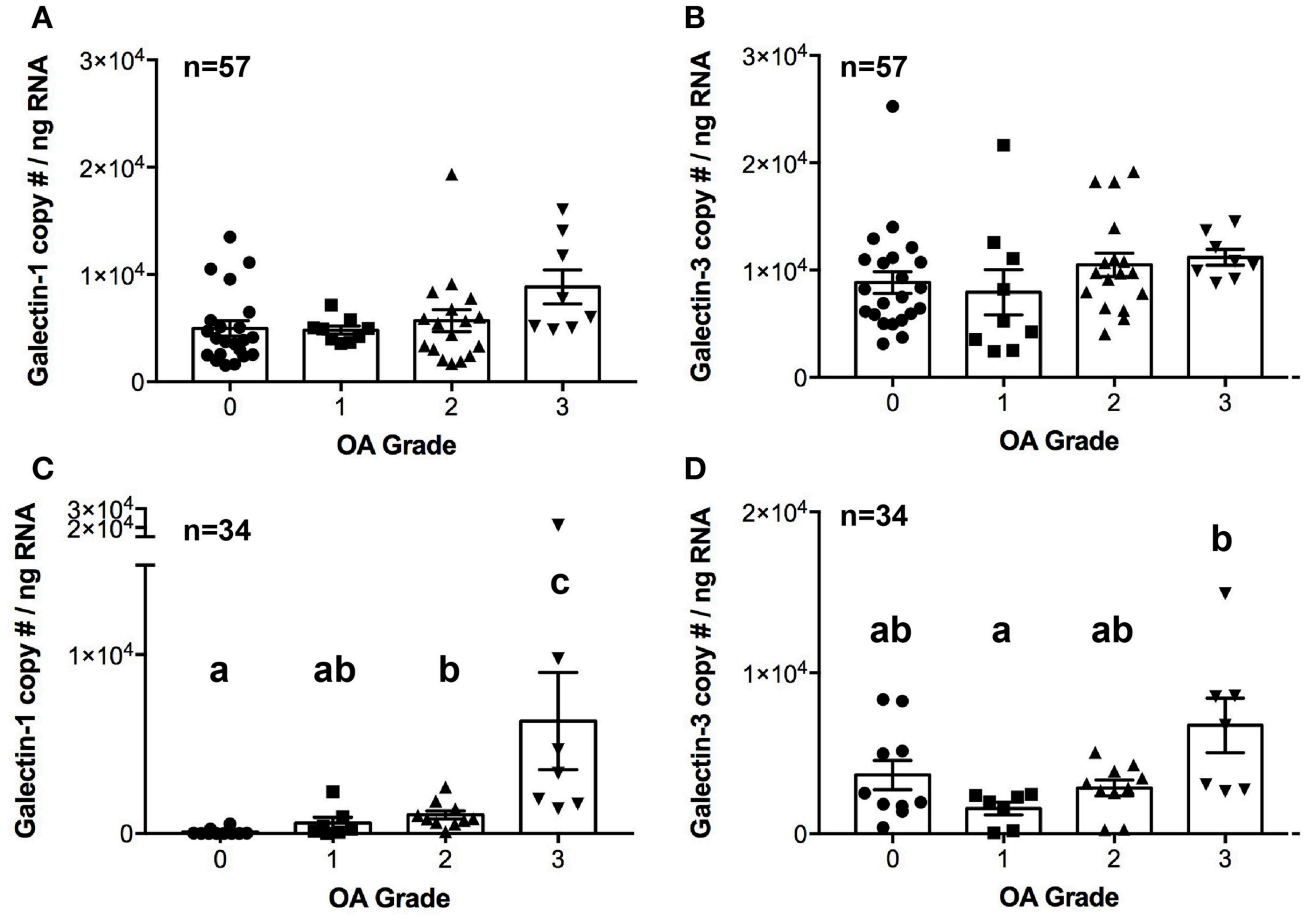
Galectin-1 and-3 copy # per ng of RNA from synovial membrane **(A,B)** or cartilage **(C,D)** tissue obtained from healthy carpal joints (0) or carpal joints with naturally occurring osteoarthritis (1–mild, 2–moderate, 3–severe OA). There were no differences in galectin-1 or-3 mRNA expression in synovial membrane tissue **(A,B)**. Cartilage galectin-1 mRNA expression increased with OA severity score (**C**, *P* = 0.0002), whereas galectin-3 mRNA expression was increased in severe OA cartilage as compared to mild OA cartilage (**D**, *P* = 0.03). Scatterplots are displayed, with bars representing mean ± S.E.M. Differing letters note statistically significant differences, *P* < 0.05.

There were no differences in galectin-1 or galectin-3 mRNA expression in synovial membrane from healthy and OA joints (Figures 3A,B). In contrast, galectin-1 mRNA expression was significantly upregulated in moderate and severe OA cartilage, with an approximately 12- and 75-fold increase as compared to healthy cartilage (Figure 3C). Galectin-1 mRNA expression was minimal in healthy (grade 0) cartilage. Galectin-3 mRNA levels were greater in severe OA as compared to mild OA (Figures 3C,D).

### Immunolocalization in experimental OA

**Figure 4:**
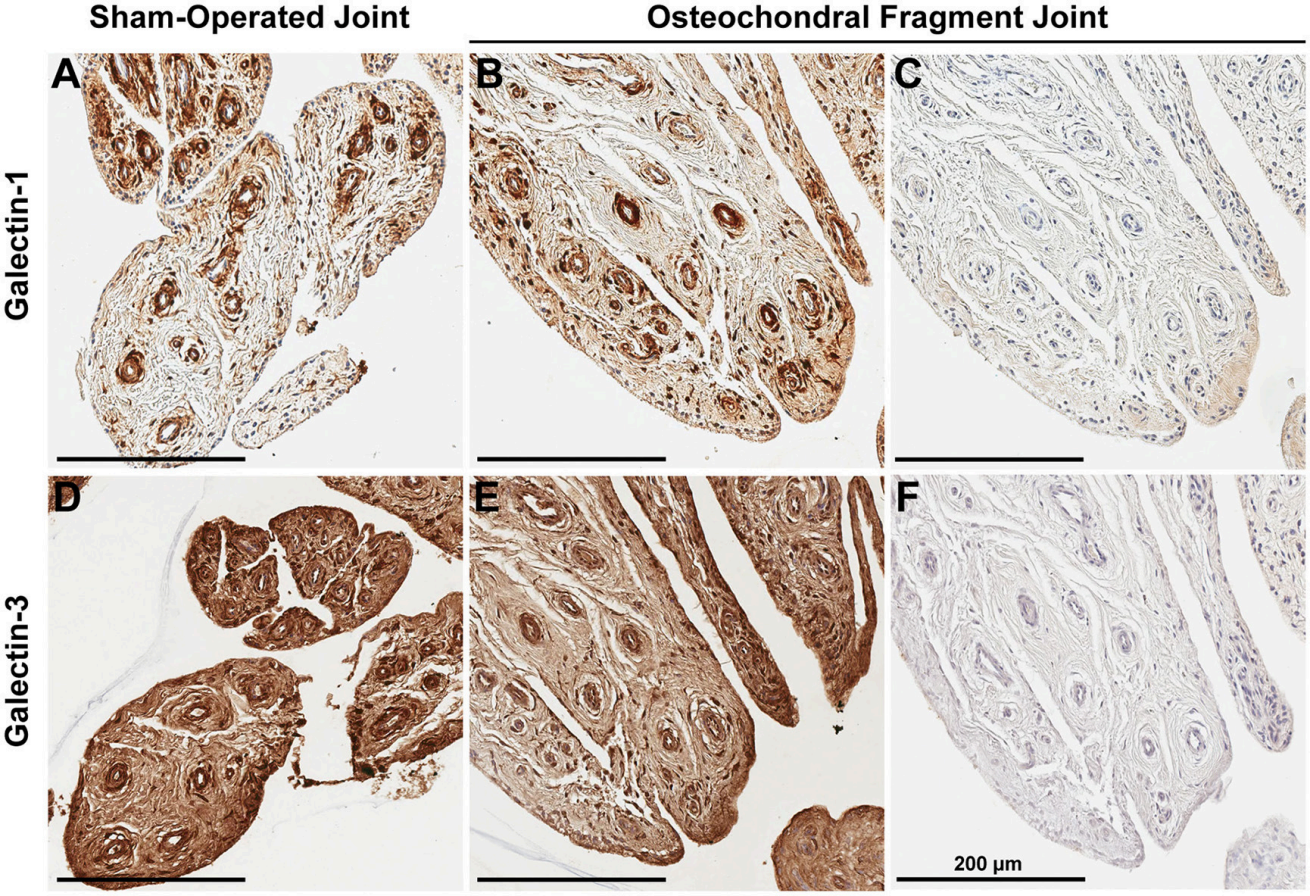
Synovial membrane galectin-1 and galectin-3 immunostaining reveals constitutive galectin expression in both sham-operated **(A,D)** and osteochondral fragment joints **(B,E)** from a representative horse 70 days post-injury. Galectin-1 and-3 staining is most prominent in perivascular regions. No differences were observed in galectin-1 or-3 staining between sham-operated and osteochondral fragment joints. Primary antibodies were omitted in **(C,F)**, revealing absence of antigen-independent staining. Scale bar: 200 μm.

**Figure 5:**
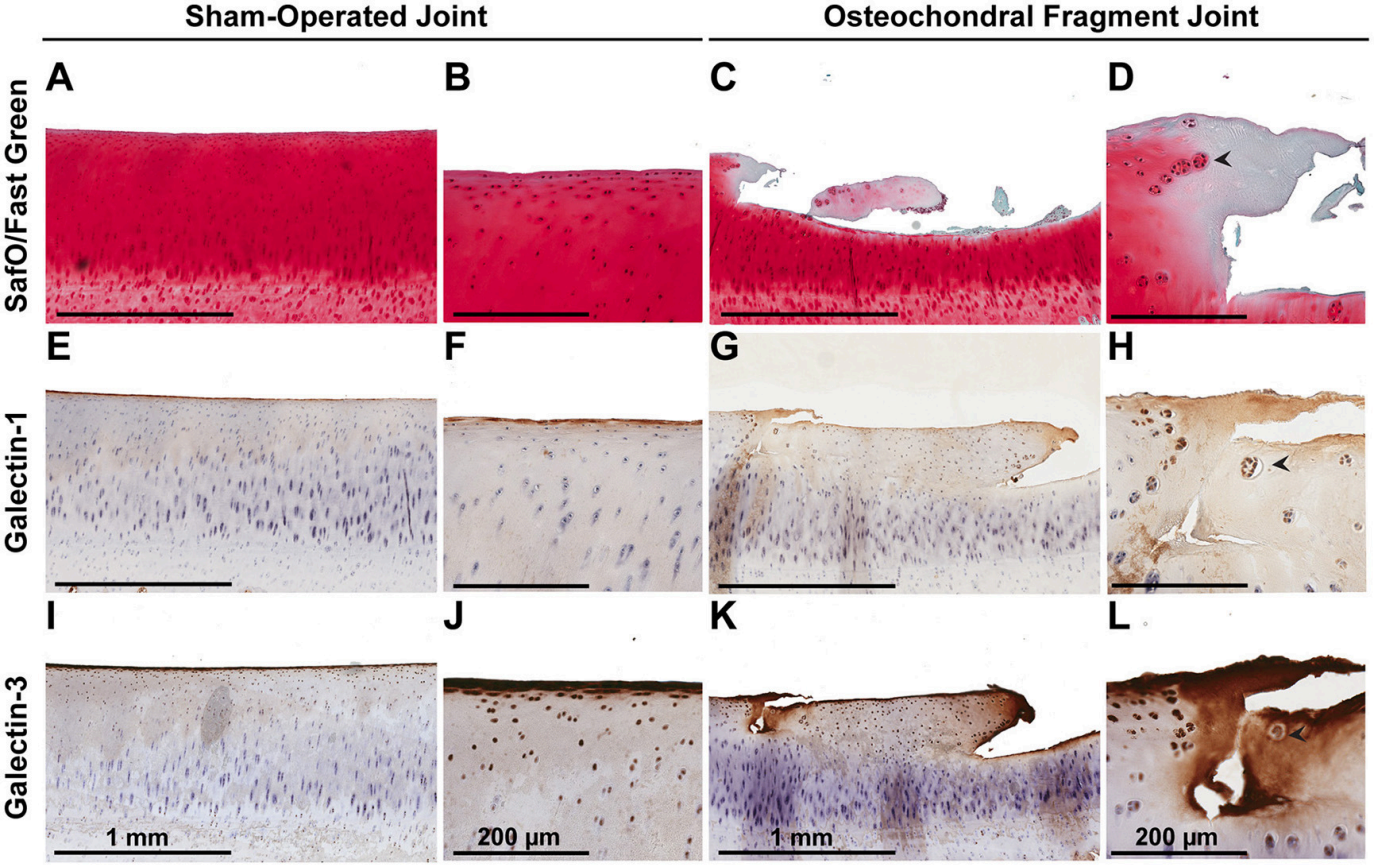
Safranin O/Fast Green **(A–D)**, galectin-1 **(E–H)**, and galectin-3 **(I–L)** immunostaining of third carpal bone cartilage from sham-operated and osteochondral fragment joints from a representative horse 70 days post-injury. Superficial zone chondrocytes and some middle zone chondrocytes stain positively for galectin-3 **(I,J)** but not galectin-1 **(E,F)** in sham-operated joints. Cartilage fibrillation, proteoglycan loss and chondrone (arrow) formation is observed in cartilage from the osteochondral fragment joint **(C,D)**. Chondrones stain positively for galectin-1 **(G,H**-arrow) and galectin-3 **(K,L**-arrow).

**Figure 6:**
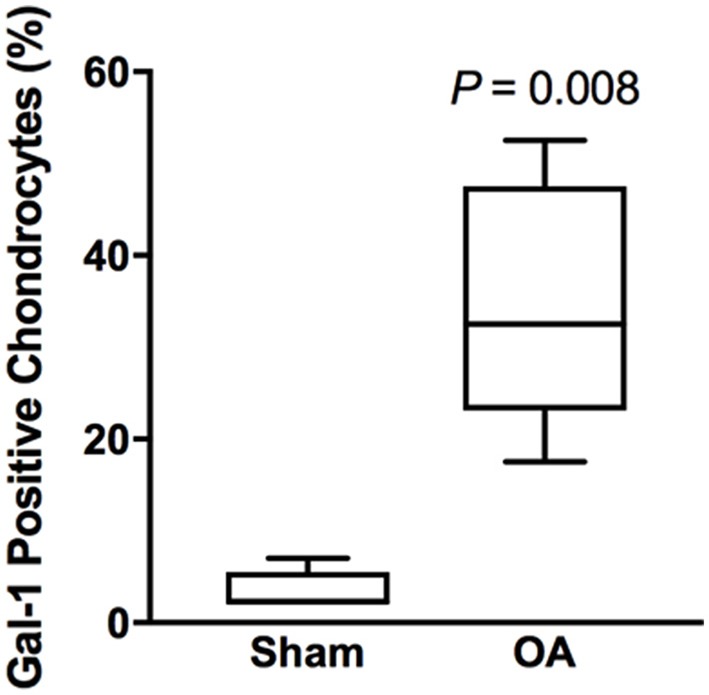
Chondrocyte galectin-1 positive immunoreactivity over the entire thickness of articular cartilage in the equine carpal fragmentation experimental model 70 days post-injury (*n* = 5). Data are presented as box-and-whiskers plots, where boxes represent the first and third quartiles, the lines within the boxes represent the median, and the lines outside the boxes represent the spread of galectin-1 scores. The *P*-value indicates the difference between injured (OA) and healthy (Sham) cartilage, produced by Wilcoxon matched-pairs signed rank test.

Synovial membrane tissue sections stained intensely for both galectin-1 and galectin-3 (Figure 4), with the most prominent immunoreaction observed in perivascular and intimal regions. Consistent with RT-qPCR data, no differences were noted in galectin synovial membrane immunostaining between sham-operated and OA joints on day 70 post-fragmentation. Weighted kappa statistics revealed moderate inter-observer agreement for both galectin-1 and-3 chondrocyte immunostaining (0.55 and 0.43, respectively). Safranin O staining was decreased in areas of partial-thickness cartilage fibrillation in the osteochondral fragment joint as compared to the sham-operated joint (Figures 5A–D). Superficial zone chondrocytes from healthy articular cartilage stained intensely for galectin-3 (Figures 5I,J), whereas galectin-1 staining was nearly absent (Figures 5E,F), consistent with gene expression data and prior immunostaining results in healthy equine cartilage (34). Galectin-1 immunostaining was increased in superficial and middle zone chondrocytes from injured cartilage (Figures 5G,H, *P* = 0.02 and 0.03, respectively) and was minimal to absent in deep zone chondrocytes in all cartilage sections. The most intense galectin-1 staining was localized to dividing chondrocytes and chondrocyte clusters (chondrones) within the superficial zone (Figure 5H, arrow). Chondrocyte galectin-1 immunoreactivity was significantly increased in cartilage from the osteochondral fragment joint as compared to the sham-operated joint (Figure 6).

There were no differences in galectin-3 chondrocyte immunostaining between healthy and injured cartilage. Superficial zone chondrocytes demonstrated intense galectin-3 expression in both healthy and injured cartilage (Figures 5I–L).

## Discussion

Galectins-1 and-3 are present in healthy equine synovial fluid and synovial tissue but increase in response to osteochondral injury. In prior work, we have demonstrated greater galectin-1 and-3 mRNA expression in healthy equine synovial membrane as compared to healthy articular cartilage (4), suggesting that the synovium may be the predominant source of synovial fluid galectins. Synovial fluid galectins-1 and-3 were elevated in experimental OA, with a transient increase in galectin-1 and a sustained increase in galectin-3. Cartilage galectin-1 mRNA expression increased with increasing OA severity, and galectin-1 immunostaining was increased in superficial and middle zone chondrocytes in injured cartilage. Whereas, galectin-3 was constitutively produced by superficial zone chondrocytes, galectin-1 immunostaining was nearly absent in healthy articular cartilage. Thus, although both galectin-1 and-3 synovial fluid concentrations are increased in joint injury, galectin-1 upregulation in injured chondrocytes appears to be a specific response to cartilage injury in horses.

### Synovial fluid galectin protein concentrations

Galectin-1 and-3 synovial fluid concentrations were increased in injured as compared to sham-operated joints. To our knowledge, this is the first study to profile serial galectin synovial fluid measurements prior to and after joint injury, revealing a transient elevation in galectin-1 after injury and a more sustained elevation in galectin-3. This longitudinal data suggests that galectins are increased in response to traumatic joint injury. Although these observations were supported by two distinct equine experimental cohorts, differences in synovial fluid galectin levels were not observed in horses with naturally occurring carpal OA. Because the OA severity grading scale for naturally occurring carpal injury involves assessment of radiographic changes, which lag behind inflammatory changes, and because the OA severity score doesn't account for the duration of injury prior to presentation, we may be missing more acute, transient elevations in galectins in horses at the time that they present to the hospital for surgical treatment.

### mRNA expression

We observed increased galectin-1 mRNA expression and immunostaining in injured equine cartilage. Interestingly, galectin-1 cartilage mRNA expression increased proportionally with the severity of arthritis. Our findings that galectin-1 mRNA expression is increased in OA cartilage and that galectin-1 immunostaining is increased in regions of focal cartilage damage coincide with prior studies in human OA cartilage (21–23). Differences in galectin-1 immunostaining were not observed in deep zone chondrocytes in the current study; however, cartilage pathology was primarily restricted to the superficial and middle zones in this equine OA model and could explain why galectin-1 immunostaining was not observed in deep zones.

Galectin-1 stimulates a network of NF-kB downstream signaling in human OA chondrocytes as demonstrated by microarray and RT-qPCR (21). Synergistic effects of galectins-1,-3, and−8 have been demonstrated in human OA chondrocytes, providing evidence for cooperativity within this galectin signaling network (24, 35). On the other hand, galectin-1 is suggested to mitigate inflammatory arthritis in rodent models by altering immune cell function, inducing apoptosis of CD4+ T cells and decreasing pro-inflammatory cytokine expression (14–17). Notably, inflammatory arthritis and post-traumatic osteoarthritis are distinct entities despite sharing some similarities, such as synovial inflammation and synovitis (36, 37).

### Immunolocalization

In addition to gene expression data demonstrating that synovial membrane is the predominant source of galectins in synovial joints, galectin immunostaining revealed prominent galectin-1 and-3 immunolocalization in synovial membrane tissue from both sham-operated and OA joints. Whereas, superficial zone chondrocytes consistently stained positively for galectin-3 in healthy cartilage, middle and deep zone chondrocytes were negative. Similar patterns of strong superficial zone chondrocyte galectin-3 staining have been previously observed in healthy equine and human cartilage (2, 34). Chondrocytes in healthy cartilage were immune negative for galectin-1. Galectin-1 positive chondrocytes were only detected in injured cartilage, especially in dividing chondrocytes or chondrones in the superficial and middle zones of injured, fibrillated cartilage.

The identification of increased galectin-3 in RA (7, 38) and juvenile idiopathic arthritis (8) has led authors to hypothesize that galectin-3 precipitates inflammatory arthritis. Elevations in serum galectin-3 concentrations have been detected in patients with early RA and correlated with MRI bone lesions 1 year later (39). However, the link between galectin-3 and OA is less clear and, to our knowledge, no human studies have documented elevations in synovial fluid galectin-3 levels preceding the development of arthritis. Data on the role of galectin-3 in experimental rodent models is conflicting. Gal-3 KO mice are predisposed to OA (6), potentially due to the protective role that galectin-3 plays in chondrocyte survival (5, 6). Galectin-3 KO mice also demonstrate decreased bone formation, increased bone resorption, accelerated trabecular bone loss and reduced bone strength as compared to wild-type mice, suggesting an important role for galectin-3 in bone remodeling and biomechanics (25). On the other hand, exogenous intra-articular galectin-3 administration promoted the development of arthritis in mice (19), and inhibition of galectin-3 through lentiviral-mediated delivery of galectin-3 shRNA ameliorated collagen-induced arthritis in rats (16). The conflicting data with respect to galectin-3 and OA may be due to the distinct functions of intracellular vs. extracellular galectin-3 and differences between inflammatory and PTOA models of arthritis. Intracellular gal-3 promotes chondrocyte survival both *in vitro* (6) and *in vivo* (6); whereas administration of exogenous, extracellular galectin-3 exacerbates inflammation (16, 18). Notably, most rodent studies investigating galectins and arthritis have focused on inflammatory models which more closely mimic RA. Horses and other large animal models are more commonly used to study PTOA and better represent the clinical scenario for translation to PTOA in humans (28, 40). Therefore, future work is needed to determine whether increased synovial fluid galectin-3 concentrations in equine PTOA are functioning to protect articular chondrocytes, promote synovial inflammation or both.

Here, we demonstrate constitutive expression of both galectin-1 and-3 in healthy synovial membrane tissue and synovial fluid, while also elucidating the time course of galectin-1 and-3 upregulation following induction of post-traumatic OA. Toegel and Weinmann et al. have suggested that both galectin-1 (21) and galectin-3 (24) promote OA through upstream regulation of NF-kB signaling in chondrocytes. Accordant with these findings, we show that galectin-1 mRNA expression and immunostaining is increased in equine OA cartilage as compared to healthy cartilage. Overall, our data suggests that galectin-1 mRNA expression and protein production is increased in injured equine articular chondrocytes, similar to injured human cartilage (21, 22). Galectin-1 appears to be more specific to articular cartilage injury in horses than galectin-3. In addition, our data suggests that synovial membrane and cartilage galectin expression patterns differ, with constitutive galectin-1 and-3 synovial membrane expression present in all sham-operated, healthy joints.

This study provides evidence for the constitutive expression and production of galectins-1 and-3 in healthy synovial joints. Whereas, galectin-3 is constitutively produced in superficial zone chondrocytes in healthy articular cartilage, both galectin-1 and-3 are expressed in healthy synovial membrane tissue. In addition, we demonstrate that synovial fluid galectins are elevated in response to PTOA and that cartilage galectin-1 expression strongly correlates with OA progression. Galectins may be potential upstream therapeutic targets in OA; however, further work is needed to clarify the mechanistic roles of galectins-1 and-3 in synovial membrane tissue, cartilage and synovial fluid in PTOA. Several small-molecule galectin antagonists and anti-galectin monoclonal antibodies are currently undergoing preclinical testing for fibrosis and cancer therapy and may have applications in other chronic inflammatory diseases, such as OA and RA (41). However, therapeutic targeting of galectins also poses significant challenges due to the context-dependent multifunctionality of galectin signaling (42) and the ability of other galectin family members to compensate for the loss of an individual galectin (35). In addition, because galectins are constitutively expressed in several tissues, off-target effects are of potential concern (43, 44). Constitutive expression of galectins in synovial fluid also suggests that there may be functional roles for galectins in healthy synovial joints, including beneficial roles in cartilage lubrication (34). Additional research is needed to clarify the functions of galectins-1 and-3 in healthy joints and in PTOA in both experimental animal models and human patients. Translational animal models will be critical for pre-clinical testing of galectin-targeted therapies for human OA.

## Data availability statement

Datasets for equine galectin genomic sequences can be found in the GENBANK repository [https://www.ncbi.nlm.nih.gov/genbank/]. The raw gene expression and ELISA data supporting the conclusions of this manuscript will be made available by the authors, without undue reservation, to any qualified researcher.

## Author contributions

HR conceived and designed the project; obtained funding for the project; acquired, analyzed and interpreted data; drafted the article; and approved the final submitted version of the article. AN obtained funding for the project, and both AN and AW contributed to the acquisition, analysis and interpretation of data; critically revised the article for important intellectual content; and approved the final submitted version of the manuscript. JS, SL, RS, and RP contributed to the acquisition, analysis and interpretation of the data; and revised and approved the final submitted version of the manuscript. SM provided statistical expertise for the analysis and interpretation of the data; critically revised the article for important intellectual content; and approved the final submitted version of the manuscript.

### Conflict of interest statement

The authors declare that the research was conducted in the absence of any commercial or financial relationships that could be construed as a potential conflict of interest.
