# Supplementary material for: Galectins-1 and-3 Increase in Equine Post-traumatic Osteoarthritis
Source: Front Vet Sci. 2018 Nov 20;5:288. doi: 10.3389/fvets.2018.00288 (PMC6256174; doi:10.3389/fvets.2018.00288)
Supplement: Supplementary file 1 [file Data_Sheet_1.PDF]

Supplementary Material

Galectins-1 and -3 Increase in Equine Post-traumatic Osteoarthritis

Heidi L. Reesink*, Alan J. Nixon, Jin Su, Sherry Liu, Ryan M. Sutton, Sabine Mann, Ashlee E. Watts, Ryan P. Peterson

*** Correspondence:** Dr. Heidi L. Reesink: hlr42@cornell.edu

Supplementary Table 1. RT-qPCR Checklist

**Sample preservation/RNA template**	**Details**
Source	Synovial membrane and articular cartilage tissue collected from the carpal joints of horses (*Equus caballus*) with healthy joints or osteoarthritic (OA) joints.
Sample preservation	Synovial membrane and articular cartilage tissues were frozen in liquid nitrogen after collection and stored at -70°C.
Sample grinding and storage	Frozen samples were crushed and ground into fine powder with a mortar & pestle and stored at -70°C.
RNA extraction and removal of genomic DNA	Total RNA was extracted from synovial membrane using the E.Z.N.A Tissue RNA Kit (Omega BioTek, Inc., Norcross, GA) or from cartilage with the RNeasy Lipid Tissue Mini Kit (QIAGEN Sciences Inc., Germantown, MD). Genomic DNA was removed by DNase I on-column digestion. Total extracted RNA was stored at -70°C.
RNA concentration and integrity	RNA concentration and quality was assessed using a Tecan Spark 10M multimode plate reader with a NanoQuant Plate™ (to detect 16 samples per assay).
**Genes assayed**	**Accession number**
Equine *Gal-1*	KY264050.1
Equine *Gal-3*	KY264051.1
Equine 18S rRNA	NR_046271.1
**Primers, probes and amplicon**	**Sequences or size**
EqGal-1 Forward	CAAGGCAGACCTGACCATCA
EqGal-1 Reverse	TGACGGCCTCCAGGTTGA
EqGal-1 probe	6-FAM/ CTGCCGGAT/ZEN/GGCTACTCGTTCAAGTTC/ IABkFQ
EqGal-1 amplicon size	77-bp
EqGal-3 Forward	TAAATTTCAACAGAGGGCATGATG
EqGal-3 Reverse	CAATGACTCTCCTGTTGTTCTCGTT
EqGal-3 probe	6-FAM/ TGCCTTCCA/ZEN/CTTTAACCCGCGCTT/ IABkFQ

EqGal-3 amplicon size	75-bp
Eq18S rRNA Forward	GGCGTCCCCCAACTTCTT
Eq18S rRNA Reverse	AGGGCATCACAGACCTGTTATTG
Eq18S rRNA probe	6-Fam/TCGAACGTCTGCCCTATCAACTTTCGAT/IABkFQ
Eq18S rRNA amplicon size	77-bp
Primer design	Primer Express Software Version 2.0 (Applied Biosystems, Foster City, CA) was used to design the forward and reverse primers and probes.
**TaqMan qRT-PCR Protocol**	
TaqMan qRT-PCR kit	TaqMan RNA-to-CT one-step kit (Applied Biosystems, Foster City, CA) was used.
RT/qPCR	RT and qPCR were carried out in the same well using theTaqMan one-step kit. Equal amounts of total RNA (10 ng per reaction) were used in a 20 µl reaction mix, and all samples were run in duplicate.
No template control (NTC)	NTCs were included in each run with each primer set.
Calculation of mRNA copy numbers	The mRNA copy numbers were calculated using the standard curves generated with serial dilutions of *E. coli*-expressed equine *Gal-1* and *Gal-3* as standards.
Data Normalization	All data were normalized to equine 18S rRNA values.
